# Explainable ML modeling of saltwater intrusion control with underground barriers in coastal sloping aquifers

**DOI:** 10.1038/s41598-025-12830-w

**Published:** 2025-08-10

**Authors:** Asaad M. Armanuos, Martina Zeleňáková, Mohamed Kamel Elshaarawy

**Affiliations:** 1 Irrigation and Hydraulics Engineering Department, Faculty of Engineering, Tanta University, Tanta, 31733 Egypt; 2 Institute of Environmental Engineering, Faculty of Civil Engineering, Technical University of Košice, 04200 Košice, Slovakia; 3 Civil Engineering Department, Faculty of Engineering, Horus University-Egypt, New Damietta, 34517 Egypt

**Keywords:** Engineering, Civil engineering

## Abstract

Reliable modeling of saltwater intrusion (SWI) into freshwater aquifers is essential for the sustainable management of coastal groundwater resources and the protection of water quality. This study evaluates the performance of four Bayesian-optimized gradient boosting models in predicting the SWI wedge length ratio (*L*/*L*_*a*_) in coastal sloping aquifers with underground barriers. A dataset of 456 samples was generated through numerical simulations using SEAWAT, incorporating key variables such as bed slope, hydraulic gradient, relative density, relative hydraulic conductivity, barrier wall depth ratio, and distance ratio. The dataset was divided into 70% for training and 30% for testing. Model performance was assessed using both visual and quantitative metrics. Among the models, Light Gradient Boosting (LGB) achieved the highest predictive accuracy, with RMSE values of 0.016 and 0.037 for the training and testing sets, respectively, and the highest coefficient of determination (R²). Stochastic Gradient Boosting (SGB) followed closely, while Categorical Gradient Boosting (CGB) and eXtreme Gradient Boosting (XGB) showed slightly higher error rates. SHapley Additive exPlanations (SHAP) analysis identified relative barrier wall distance and bed slope as the most influential features affecting model predictions. To support practical application, an interactive graphical user interface (GUI) was developed, allowing users to input key variables and easily estimate *L*/*L*_*a*_ values. Finally, the best-performing model was validated against the Akrotiri coastal aquifer in Cyprus, a realistic benchmark case derived from numerical simulations. The model’s predictions showed strong agreement with reference results, achieving an RMSE of 0.04, thereby confirming its practical applicability. This study highlights the potential of interpretable, optimized ML models to enhance SWI prediction and support informed decision-making in coastal aquifer management.

## Introduction

Most of the drinkable water supply in both urban and rural regions comes from groundwater^1^. In addition to the global shortage of drinkable water, population increase, industrialization, and overexploitation are the main causes of the significant decline in groundwater quality over the past few decades^2^. Over the past few decades, providing clean, drinkable water has grown more difficult, particularly in poorer nations^3^. Over 50% of urban people and over 90% of rural families rely on groundwater resources for their drinking water. Additionally, coastal areas’ groundwater aquifers are vulnerable to saltwater intrusion through a variety of pathways, including faults and lineaments that expand toward the ocean^4^.

Over 40% of the population lives within 100 km of the ocean, making this issue worse in coastal areas^5–8^. Both natural and man-made factors, including climate change, surface and subsurface topography, groundwater flow themes, tidal variants, hydraulic gradient, sea-level rise, saltwater intrusion, overuse of groundwater, and coastal erosion, contribute to saline/saltwater invasion in fresh coastal groundwater systems^9–11^. When fresh water is extracted from these aquifers, enhanced hydraulic gradients allow saltwater to migrate onshore. High tides, tsunamis, and cyclones further upset the balance between fresh and salty water^12^. The groundwater in coastal regions is therefore particularly vulnerable to salinization^13^. In regions where freshwater aquifers are hydraulically associated with saltwater, salinization or saltwater intrusion into coastal aquifers has emerged as a significant global concern^14^.

Armanuos et al.^15^ and Todd^16^ compiled several strategies to control or prevent SWI in coastal aquifers. These tactics can be summed up as: (1) reducing the abstraction of groundwater^17,18^; (2) setting up artificial recharge wells and spreading basins for artificial recharge^19–21^; (3) regionally injecting freshwater in the coastal zone^22^; (4) extracting saltwater^23^; (5) erecting artificial barriers^19,24–30^; (6) combination approaches^19,31,32^; and (7) land reclamation^33,34^.

The effects of employing cutoff walls to prevent saltwater intrusion were investigated by Anwar^35^. Using a cutoff wall, an analytical solution was developed in accordance with the sharp interface technique to identify the position of the saltwater interface^36^. To find out how well barrier walls work to control SWI, several researchers have conducted laboratory tests^24^. The usage of subterranean barrier walls with varying embedding depths and positions to control SWI was examined experimentally by Luyun et al.^37^. The findings demonstrated that SWI retreated, and the repulsion ratio of the saltwater invasion (R) increased as the subterranean barrier wall was embedded deeper into the aquifer and closer to the saltwater side.

In order to control SWI in stratified aquifers, Abdoulhalik and Ahmed^38^ conducted a laboratory investigation and numerical analysis. A contemporary hybrid flow barrier that includes a cutoff wall with a semi-pervious dam was created by Abdoulhalik et al.^38^. The created wall achieved a large SWI repulsion ratio while maintaining SWI transit to the ocean side. In order to manage the advancement of SWI, Armanuos et al.^19^ conducted research experiments to examine the efficacy of employing an underground barrier wall, freshwater injection through wells, and a combination of approaches.

The impact of employing barrier walls to regulate SWI in sloped unconfined aquifers was investigated by Armanuos et al.^27^ using SEAWAT software. Three distinct bed slopes are taken into consideration for the sloping unconfined aquifer. The results verified that the saltwater interface was compelled to travel seaward, and the repulsion ratio (R) rose as the ratio of flow barrier depth increased. In the three bed-sloping scenarios, a higher R-value was obtained by embedding the barrier wall to a *d*_*b*_/*d* value of more than 0.4. The SWI’s withdrawal was aided by the barrier wall’s deeper installation close to the saltwater side. Emara et al.^30^ assessed the effect of an inclined cutoff wall to regulate saltwater intrusion in diverse coastal aquifers using SEAWAT software. Using both experimental and numerical simulations, the study investigated the dynamics of SWI under different cutoff-wall inclination degrees and depths, in addition to aquifer heterogeneity. By classifying various hydraulic conductivity layers into two different cases: high *K* above low *K* (case *H*/*L*) and low *K* above high *K* (case *L*/*H*)-stratified aquifers were produced. For cutoff-wall depth ratios up to 0.623, the highest repulsion ratio of SWI wedge length was noted at an inclination angle of 76.0°. For every aquifer under study, the maximum repulsion ratio changed to an angle of 63.4° when the depth ratio rose to 0.811.

Chang et al.^39^ investigated the impact of cutoff walls on SWI repulsion for both homogeneous and stratified unconfined aquifers using SEAWAT software. The findings showed that cutoff walls boosted the inland groundwater level, creating a huge hydraulic gradient to resist SWI by creating a big groundwater level differential along two of the wall’s sides. They concluded that building a cutoff wall might outcome in higher inland freshwater hydraulic head and a rapid freshwater velocity through raising inland freshwater influx. The saltwater wedge was pushed seaward by a significant hydraulic pressure caused by the high inland freshwater hydraulic head.

In order to examine the effects of mixed physical barriers (MPB) for saltwater removal in coastal heterogeneous aquifers, Emara et al.^29^ used SEAWAT software. In a laboratory-scale aquifer, the effectiveness of MPBs was contrasted with that of individual physical barriers. Two stratified aquifers (HLH and LHL) and a homogenous reference aquifer (H) made up the three distinct configurations that were duplicated. The findings show that MPBs effectively shorten the saltwater penetration length in the circumstances under investigation. In every instance, the penetration length was reduced by up to 65%.

Despite increasing interest in data-driven hydrological modeling, the application of advanced ML techniques to SWI wedge length prediction in coastal sloping aquifers with underground barriers remains underexplored. Existing studies primarily rely on analytical or numerical models, with limited use of ML to capture the nonlinear interactions among key variables such as groundwater head, flow rates, aquifer geometry, and barrier configurations. Moreover, where ML has been applied, models often lack interpretability, hindering their integration into engineering practice. Few studies have employed explainable AI tools like SHAP to quantitatively assess input feature contributions, and comprehensive validation protocols, such as cross-validation, uncertainty quantification, and error distribution analysis, are rarely implemented.

Furthermore, there is a clear lack of user-oriented tools to bridge the gap between ML model development and operational decision-making. This study addresses these deficiencies by combining Bayesian-optimized gradient boosting models with SHAP-based interpretation and embedding the results into a user-friendly GUI to enhance the practical utility of ML in SWI management. Finally, to evaluate real-world applicability, the best-performing model was validated against the Akrotiri coastal aquifer in Cyprus. This demonstrates the strong predictive capability and practical relevance of the proposed modeling approach.

## Methodology

**Fig. 1:**
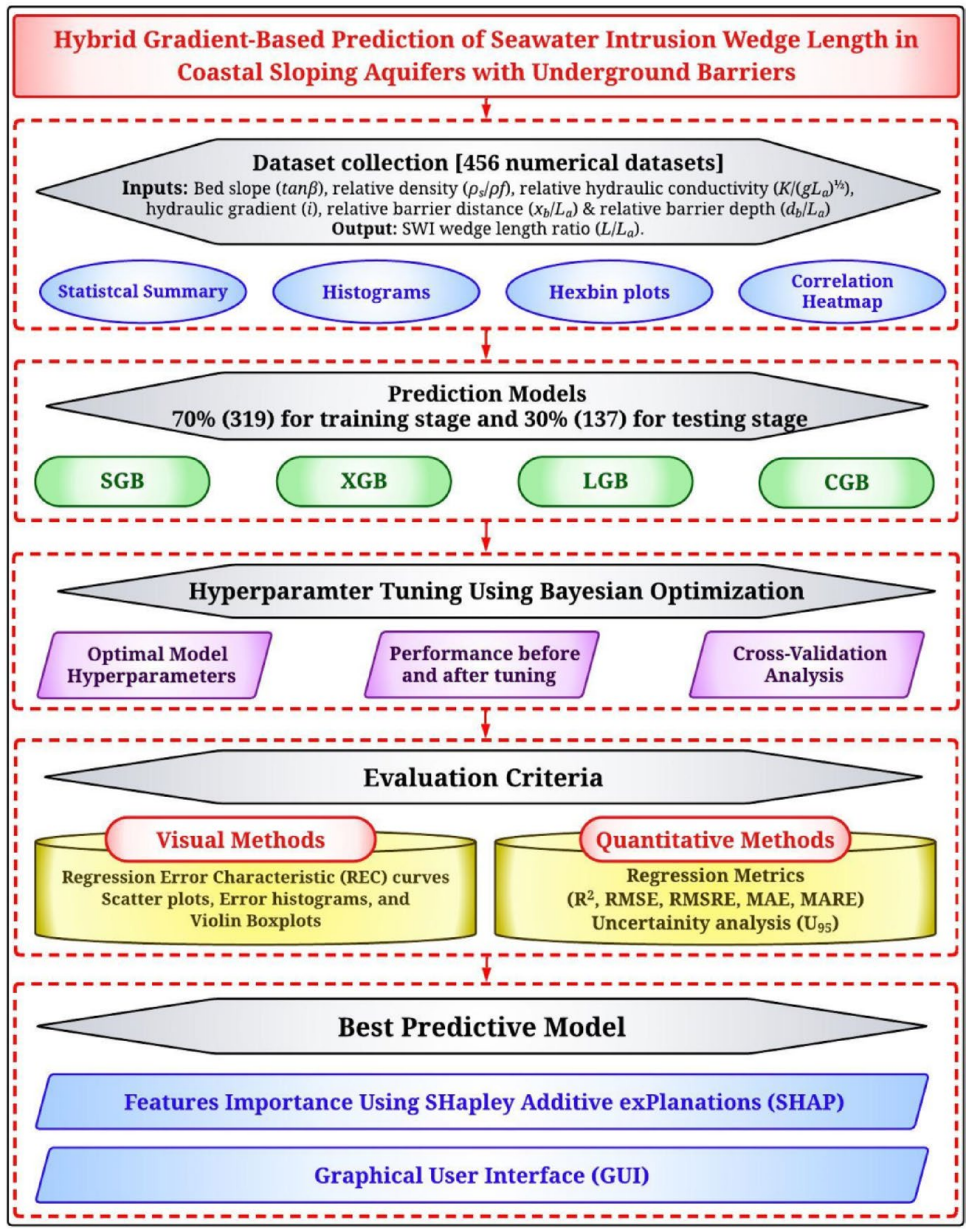
Adopted methodological flowchart.

Figure 1 outlines the methodology for predicting *L*/*L*_*a*_ in coastal sloping aquifers with underground barriers using Bayesian-optimized gradient boosting models. A dataset of 456 numerical samples, containing key hydraulic parameters as inputs and *L*/*L*_*a*_ as the target, was compiled and explored using statistical summaries, histograms, hexbin plots, and correlation heatmaps. Four advanced ML models: SGB, XGB, LGB, and CGB were developed to capture non-linear patterns in the data. Bayesian optimization was used for hyperparameter tuning, with cross-validation ensuring model generalizability. Model performance was assessed using both visual tools (residual error curves, scatter plots, violin plots) and quantitative metrics. Uncertainty analysis was conducted to evaluate prediction confidence. SHAP analysis was applied to the best-performing model to interpret feature importance. Finally, a user-friendly GUI was created to support practical, transparent application of the model for real-world SWI prediction.

### Database Collection

**Fig. 2:**
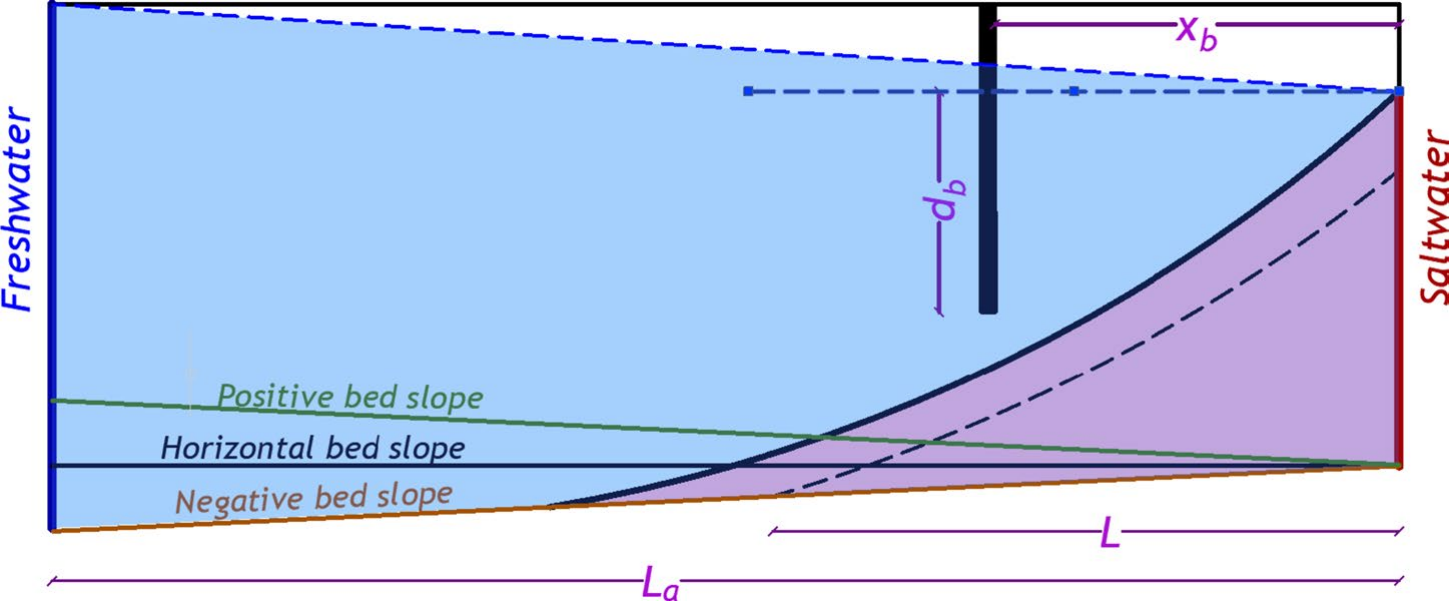
Schematic of an underground barrier wall located in an unconfined coastal aquifer with the investigated parameters.

This investigation employs a dataset comprising 456 numerical scenarios derived from the work of Armanuos et al.^27^ aimed at evaluating the effectiveness of subterranean cutoff walls in mitigating SWI within unconfined coastal aquifers featuring sloping beds. This dataset serves as the foundational input for the development and validation of predictive modeling approaches. Figure 2 illustrates a conceptual representation of how subsurface barriers are utilized to manage SWI under conditions of positive, horizontal, and negative aquifer slopes. The analysis encompasses several key variables: freshwater density (*ρ*_*f*_), saltwater density (*ρ*_*s*_), gravitational acceleration (*g*), saturated hydraulic conductivity of the unconfined coastal aquifer (*K*), hydraulic gradient (*i*), the horizontal distance of the cutoff wall from the shoreline (*x*_*b*_), the depth of the barrier wall (*d*_*b*_), the length of the saltwater intrusion wedge following the implementation of the barrier (*L*), and the total aquifer length (*L*_*a*_).

By using dimensional analysis, Eq. (1) is derived as presented below:

1 $${\phi _1}\left( {{\rho _s},{\rho _f},g,i,K,{x_b},{d_b},tan\beta ,L,{L_a}} \right)=0$$

In Eq. (1), the term *ϕ*_1_​ represents a functional expression. Utilizing *ρ*_*f*_, *L*_*a*_, and *g* as the set of repeating variables, the total number of influencing variables in the analysis is identified as n equals 10. The system incorporates three fundamental physical dimensions, namely length (L), mass (M), and time (T), as defined by the Buckingham π theorem^40^. As a result, the dimensional analysis yields a total of seven independent, dimensionless groups (π-terms), which are explicitly formulated in Eqs. (2) and (3).

2 $$\:{\varphi\:}_{2}\left({\pi\:}_{1},{\pi\:}_{2},{\pi\:}_{3},{\pi\:}_{4},{\pi\:}_{5},{\pi\:}_{6},{\pi\:}_{7}\right)=0$$

3 $$\:{\varphi\:}_{2}\left(\frac{{\rho\:}_{s}}{{\rho\:}_{f}},\frac{K}{\sqrt{g{L}_{a}}},tan{\upbeta\:},i,\frac{{x}_{b}}{{L}_{a}},\frac{{d}_{b}}{{L}_{a}},\frac{L}{{L}_{a}}\right)=0$$

where *ϕ*_2_ is a functional symbol. Notably, *ρ*_*f*_, *g*, and *L*_*a*_ are considered constant, as demonstrated in^21,27^. Accordingly, Eq. (4) is the simplified formula used for estimating *L*/*L*_*a*_. It can be expressed as follows:

4 $$\:\frac{L}{{L}_{a}}={{\upvarphi\:}}_{3}\left(tan{\upbeta\:},i,\frac{{\rho\:}_{s}}{{\rho\:}_{f}},\frac{K}{\sqrt{g{L}_{a}}},\frac{{x}_{b}}{{L}_{a}},\frac{{d}_{b}}{{L}_{a}}\right)$$

where *ϕ*_3_ is a functional symbol, *tan*β is the bed slope, *i* is the hydraulic gradient, *ρ*_*s*_/*ρ*_*f*_ is the relative density, *K*/(*gL*_*a*_)^½^ is the relative hydraulic conductivity, *x*_*b*_/*L*_*a*_ is the relative barrier wall distance, and *d*_*b*_/*L*_*a*_ is the relative barrier wall depth.

#### *Descriptive statistics*

**Fig. 3:**
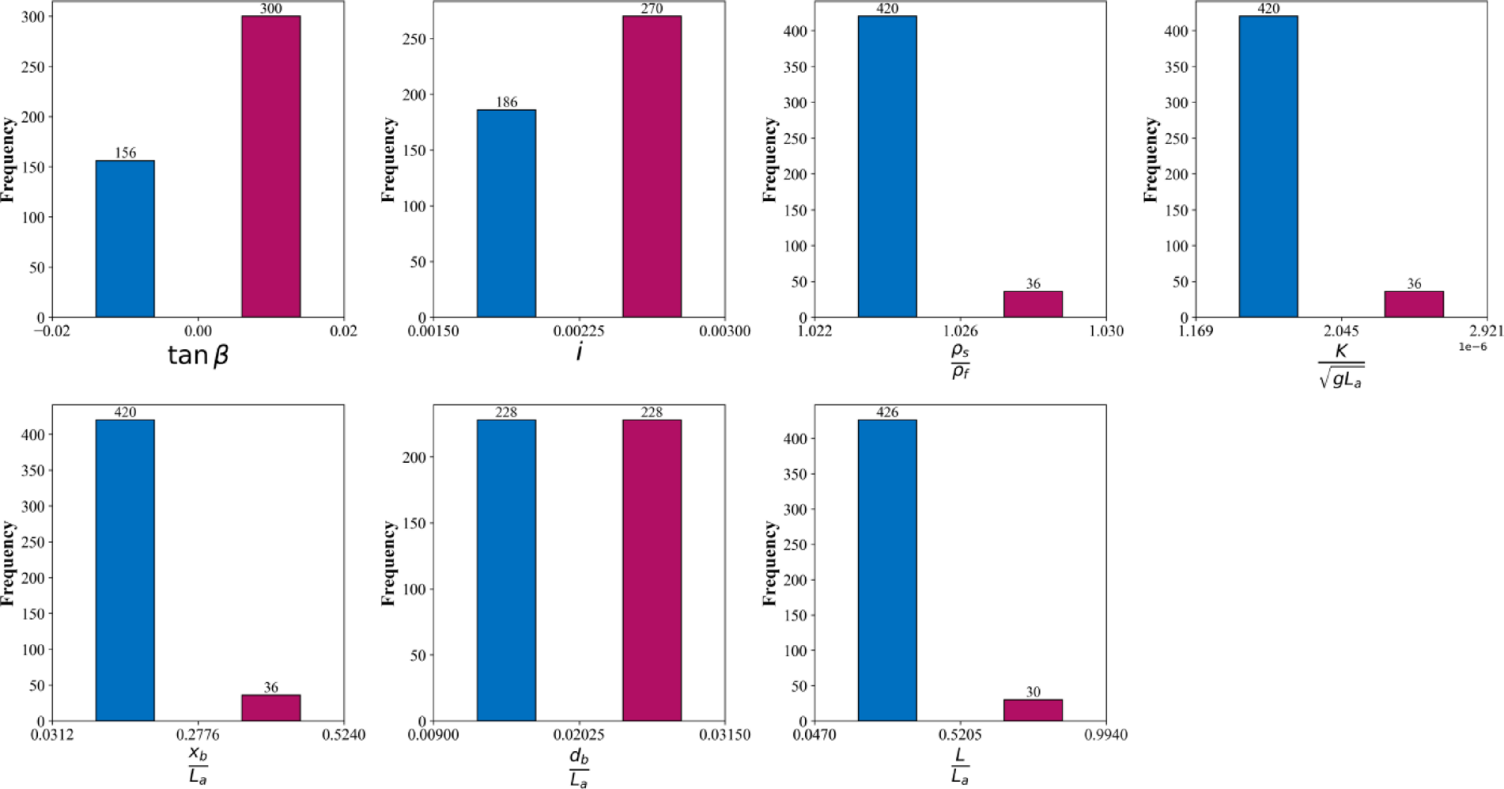
Histograms of each variable in the collected dataset.

The statistical summary provided in Table 1 and the histograms in Fig. 3 offer an insightful overview of the dataset’s variables and their distributions. It can be observed that the variables exhibit a wide range of values, reflecting the variability within the dataset. For instance, the minimum and maximum values of *tanβ* range from − 0.02 to 0.02, with a mean of zero, indicating a symmetric distribution around zero. Similarly, *i* shows a narrow range between 0.0015 and 0.0030, with a mean value close to the median of 0.0025, suggesting a relatively uniform spread.

**Table 1:** Statistical summary of the collected database.

Statistics	*tanβ*	*i*	*ρ*_*s*_/*ρ*_*f*_	*K*/(*gL*_*a*_)^1/2^	*x*_*b*_/*L*_*a*_	*d*_*b*_/*L*_*a*_	*L*/*L*_*a*_
Minimum	− 0.0200	0.0015	1.0220	1.17E-06	0.0312	0.0090	0.0470
Maximum	0.0200	0.0030	1.0300	2.92E-06	0.5240	0.0315	0.9940
Mean	0.0000	0.0021	1.0252	1.31E-06	0.1336	0.0203	0.2801
Median	0.0000	0.0025	1.0250	1.17E-06	0.1099	0.0203	0.2560
Standard Deviation	0.0161	0.0005	0.0012	4.12E-07	0.1021	0.0077	0.1553

*ρ*_*s*_/*ρ*_*f*_ demonstrates a small standard deviation of 0.0012 around its mean value of 1.0252. This indicates that the dataset is centered near the mean with little variation. On the other hand, *K*/(*gL*_*a*_)^½^ has a minimum value of 1.17 × 10^− 6^ and a maximum of 2.92 × 10^− 6^, with a standard deviation of 4.12 × 10^− 7^, reflecting a consistent but narrow range of values. The variables *x*_*b*_/*L*_*a*_ and *d*_*b*_/*L*_*a*_ exhibit larger variations, particularly *x*_*b*_/*L*_*a*_, with a standard deviation of 0.1021, suggesting heterogeneity in the dataset. Similarly, *L*/*L*_*a*_ ranges between 0.0470 and 0.9940, with a mean of 0.2801 and a standard deviation of 0.1553, indicating a significant variation in the observed output.

The histograms in Fig. 3 visually confirm the statistical trends highlighted in Table 1. The distributions of *tanβ* and *i* are symmetric, aligning well with their mean and median values being nearly identical. *ρ*_*s*_/*ρ*_*f*_ appears to be narrowly distributed around its mean, with most values concentrated near 1.025, corroborating the small standard deviation observed in Table 1. For *K*/(*gL*_*a*_)^½^, the histogram reveals a skewed distribution, with a majority of values clustering near the lower bound, consistent with the narrow range described in the statistical summary. The histograms for *x*_*b*_/*L*_*a*_ and *d*_*b*_/*L*_*a*_ indicate that these variables are more diverse, showing multimodal tendencies and larger spreads, which explains their higher standard deviations. Finally, the histogram of *L*/*L*_*a*_ exhibits a wide range, emphasizing the diversity in the response variable, which is consistent with the significant standard deviation noted in the table.

Together, Table 1 and Fig. 3 demonstrate that the dataset is well-structured with clear patterns and variability across the variables. The statistical consistency between the table and the histograms provides confidence in the dataset’s quality and highlights the diversity and complexity captured by the parameters. This balance of variation and structure suggests that the dataset is suitable for modeling and analysis to investigate the relationships between the input parameters and the response variable.

#### *Correlation analysis*

**Fig. 4:**
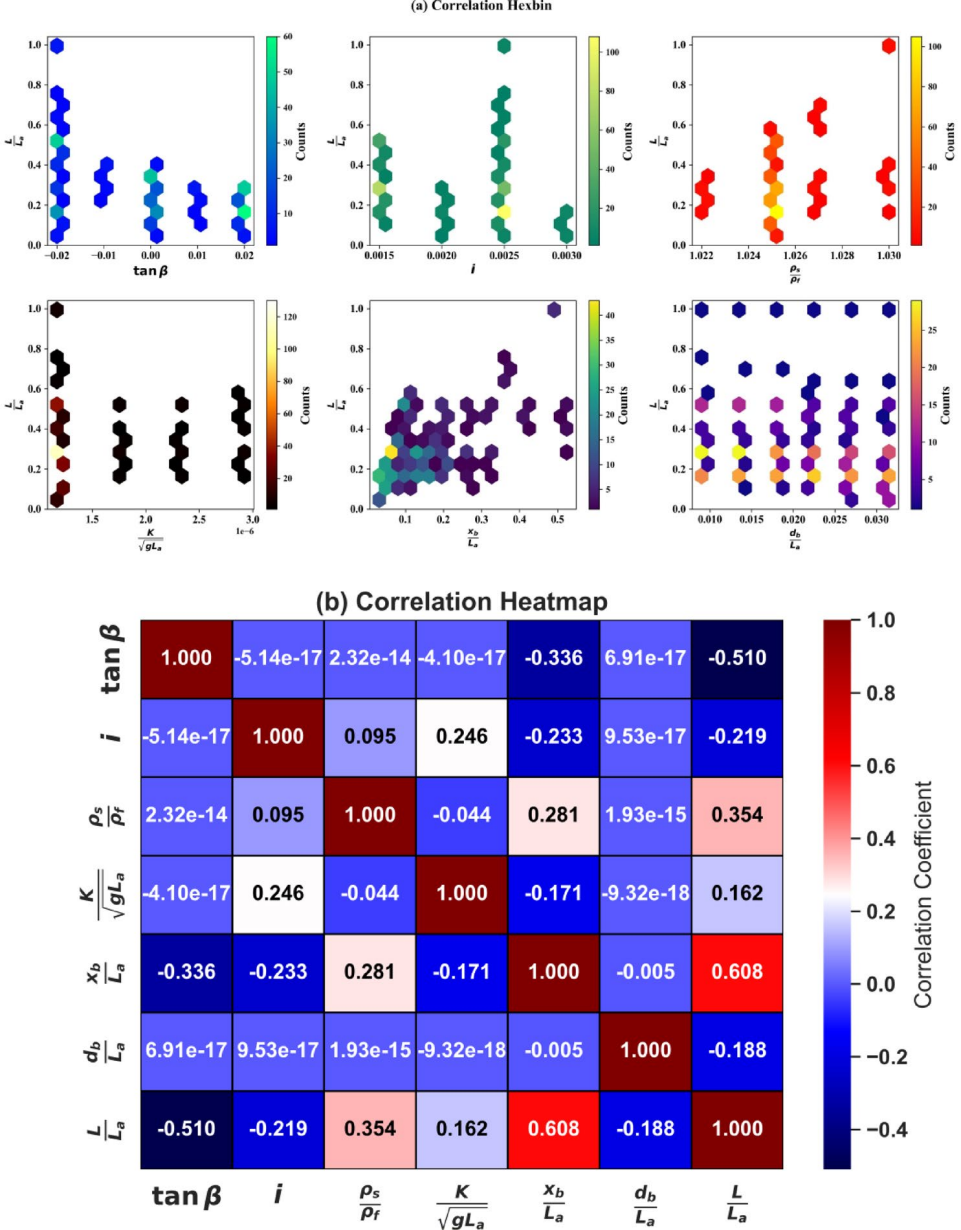
Correlation relationship between inputs and the output (**a**) hexbin plots and (**b**) heatmap.

The provided visualizations in Fig. 4 offer complementary insights into the relationships between the input parameters and the output variable. The hexbin plots in Fig. 4a highlight clustering patterns between the input variables and *L*/*L*_*a*_. For *tanβ*, the output values appear uniformly distributed across the range of the input, with no strong trend observed. In contrast, *i* shows some clustering of higher output values around specific regions, though the overall relationship remains weak. *ρ*_*s*_/*ρ*_*f*_ exhibits more distinct groupings, with higher counts corresponding to specific density ratios, indicating a potential weak to moderate correlation. Similarly, *K*/(*gL*_*a*_)^½^ demonstrates clustering patterns but no clear monotonic relationship. *x*_*b*_/*L*_*a*_ and *d*_*b*_/*L*_*a*_ reveal more structured relationships with *L*/*L*_*a*_, particularly for *x*_*b*_/*L*_*a*_, where higher values of the output are associated with larger input values.

The heatmap in Fig. 4b provides a quantitative assessment of these relationships through Pearson correlation coefficients. It confirms that *x*_*b*_/*L*_*a*_ has the strongest positive correlation with *L*/*L*_*a*_ (0.608), indicating a moderately strong relationship. This is consistent with the hexbin plot, where structured clusters were observed. Additionally, *ρ*_*s*_/*ρ*_*f*_ shows a weaker positive correlation (0.354), which aligns with the clustering pattern seen in its corresponding hexbin plot. The remaining variables *tanβ*, *i*, *K*/(*gL*_*a*_)^½^, and *d*_*b*_/*L*_*a*_ exhibit weak or negligible correlations with *L*/*L*_*a*_, with correlation coefficients close to zero. Notably, *tanβ* has the strongest negative correlation with the output (− 0.510), suggesting a slight inverse relationship.

### Gradient boosting models

SGB, also known as Gradient Boosted Regression Trees (GBRT), is a highly versatile and powerful machine learning algorithm widely recognized for its effectiveness in handling structured or tabular data^41^. It builds upon the traditional gradient boosting framework, which sequentially combines weak learners, typically decision trees, to minimize a specified loss function. SGB enhances this process by introducing a layer of randomness during training, which not only improves the algorithm’s ability to generalize across datasets but also reduces the likelihood of overfitting^42^.

XGB is a powerful and efficient evolution of traditional gradient boosting methods^43^. It constructs an ensemble of decision trees in a step-by-step manner, with each tree designed to reduce the prediction errors made by the previous ones by optimizing a defined loss function. XGB enhances model robustness by incorporating regularization strategies that help control overfitting. Additionally, it supports parallel computation, allowing for faster training on large-scale datasets. Due to its adaptability, speed, and high accuracy, XGB has gained widespread use across various machine learning tasks and has become a standout performer in competitive data science environments.

LGB is an efficient and scalable gradient boosting algorithm that focuses on improving the performance and speed of traditional gradient boosting models. It utilizes a novel histogram-based approach to bin continuous features, reducing memory usage and speeding up training^44^. LGB is designed for large datasets and high-dimensional data, making it particularly useful for real-world applications. It provides competitive accuracy with lower computational costs compared to other boosting algorithms. Additionally, LGB supports categorical features directly, eliminating the need for one-hot encoding^45^.

CGB is an advanced gradient-boosting technique designed to efficiently handle categorical variables, eliminating the need for complex encoding procedures^46^. It uses an innovative approach to encode categorical variables and employs techniques like ordered boosting to avoid overfitting. The key hyperparameters that impact the model’s performance include learning_rate, which determines the size of updates during training, and iterations, which specify the number of boosting rounds. The depth of the trees, controlled by the depth parameter, balances the ability to capture complex patterns with the risk of overfitting^47^.

Tuning hyperparameters plays a vital role in maximizing the effectiveness of ML models^48^. Conventional approaches such as grid search and random search often fall short in efficiency, as they rely on exhaustive or uninformed sampling strategies that can be both time-intensive and computationally costly. Alternatively, Bayesian optimization offers a smarter solution by leveraging previous evaluation results to strategically guide the search process, significantly cutting down the number of iterations required. To enhance model generalization and minimize the risk of overfitting, this study employs a combined strategy of Bayesian optimization with 5-fold cross-validation (BO + 5CV), allowing for consistent performance assessment across varied data splits and yielding more robust, dependable predictions^49^.

### Evaluation criteria

The assessment of the proposed predictive models is performed through an integrative framework that combines both graphical tools and quantitative performance indicators to ensure a rigorous evaluation^50^. Scatter diagrams are employed to visually explore the degree of agreement between observed and estimated outcomes, offering an intuitive means to identify the accuracy and any systematic biases in the predictions. Furthermore, Regression Error Characteristic (REC) curves are utilized to evaluate the predictive capability of the models across a spectrum of permissible error margins, thereby illustrating the proportion of forecasts that fall within specific tolerance thresholds.

To quantitatively characterize the models’ performance, several statistical indicators are applied. Table 2 presents the mathematical formulations used to compute the regression metrics serving to comprehensively capture aspects of predictive accuracy, consistency, and bias^51^. Moreover, prediction uncertainty is quantified using the 95% uncertainty interval (U_95_), derived by integrating the standard deviation and RMSE. This measure provides a statistical indication of the confidence level associated with the model outputs, reflecting the extent of variability and the reliability of the predictions. Altogether, this multi-criteria evaluation methodology delivers an in-depth appraisal of model performance, revealing both its strengths and limitations across various analytical dimensions.

**Table 2:** Selected metrics for evaluating the models’ performance.

Regression metric	Remarks
$\:{\text{R}}^{2}=1-\frac{{\sum\:}_{i=1}^{n}{\left({y}_{i}-\widehat{{y}_{i}}\right)}^{2}}{{\sum\:}_{i=1}^{n}{\left({y}_{i\:}-\stackrel{-}{y}\right)}^{2}}$	*n* represents the number of data points in the dataset. $\:{y}_{i}$ and $\:\widehat{{y}_{i}}$ denote the actual and predicted values for the iiith instance, respectively. $\:\stackrel{-}{y}$ is the mean of the actual values. $\:\stackrel{-}{\widehat{y}}\:$ is the mean of the predicted values. *σ* represents the standard deviation of the predicted values.
$$\:\text{R}\text{M}\text{S}\text{E}=\sqrt{\frac{{\sum\:}_{i=1}^{\:n}{\left({y}_{i}-\widehat{{y}_{i}}\right)}^{2}}{n}\:}$$	
$$\:\text{R}\text{M}\text{S}\text{R}\text{E}=\sqrt{\frac{1}{n}{\sum\:}_{i=1}^{\:n}{\left(\frac{{y}_{i}-\widehat{{y}_{i}}}{{y}_{i}}\right)}^{2}}$$	
$$\:\text{M}\text{A}\text{E}=\frac{{\sum\:}_{i=1}^{\:n}\left|{y}_{i}-\widehat{{y}_{i}}\right|}{n}$$	
$$\:\text{M}\text{A}\text{R}\text{E}=\frac{\sum\:\left|\frac{{y}_{i}-\widehat{{y}_{i}}}{{y}_{i}}\right|}{n}$$	
$$\:{\text{U}}_{95}=1.96\sqrt{{RMSE}^{2}+{{\upsigma\:}}^{2}}$$	

### Feature interpretability and importance

To understand how each input variable influences the model’s predictions, this study employed SHapley Additive exPlanations (SHAP). SHAP is a widely used interpretability technique that quantifies the individual contribution of each feature to a given prediction^52–54^. It breaks down the model’s output into additive components linked to each input, allowing for both global and instance-specific explanations.

Unlike traditional statistical tools such as Pearson correlation, which only measure linear relationships between variables, SHAP captures complex, non-linear interactions and the combined effects of multiple inputs. Pearson coefficients are useful for identifying general trends, but they fail to account for interactions or feature importance within a trained model. SHAP overcomes these limitations by offering a more detailed and model-aware perspective, which is particularly valuable when dealing with the non-linear behavior typical in hydrogeological systems.

Visual tools such as SHAP summary plots provide a ranked overview of feature influence, while dependence and interaction plots help reveal threshold effects and multi-feature dependencies. This interpretability framework enhances transparency and helps validate the ML model’s internal logic, making it easier for researchers and practitioners to trust and apply its predictions in real-world SWI control scenarios.

###  Case study validation

**Fig. 5:**
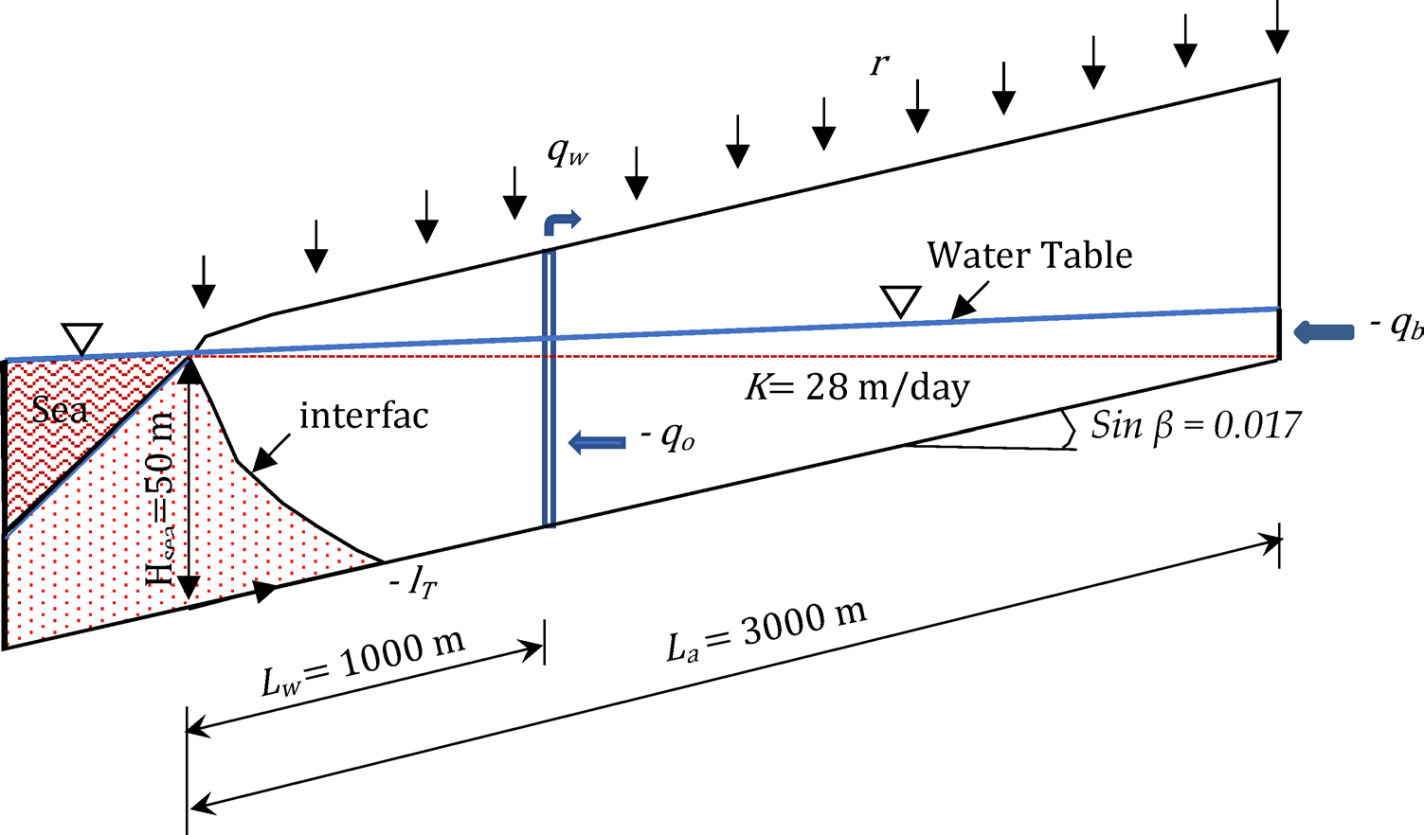
Conceptual profile of the Akrotiri coastal aquifer, Zakaki area, Cyprus.

To assess the real-world applicability of the proposed ML models, a numerical case study of the Akrotiri coastal aquifer, located in the Zakaki area of Cyprus, was used for validation. This aquifer has been extensively studied in the context of SWI, originally modeled analytically by Koussis et al.^55^ and later simulated using the SEAWAT numerical code by Armanuos et al.^27^. The SEAWAT-based simulations incorporated realistic aquifer parameters and SWI behavior under various underground barrier wall configurations. This makes the Akrotiri aquifer a suitable benchmark for testing the accuracy and generalizability of ML predictions. The aquifer’s location and conceptual layout are illustrated in Fig. 5.

It is positioned along the southern coast of Cyprus and features a positively sloped, unconfined coastal structure. The aquifer follows a typical inland-to-coast orientation and includes a known saltwater interface. The total modeled length of the aquifer was 3000 m, with a slope of approximately 1.7%, and a uniform aquifer depth of 100 m at the coastal boundary. A hydraulic conductivity of 28 m/day and a specific yield of 0.2 were used in the SEAWAT simulations. Freshwater and saltwater densities were 988.275 kg/m³ and 1024 kg/m³, respectively, and the inland flow boundary was set to 314 m³/year/m. Recharge was applied at a rate of 83 mm/year. Among the tested cases, five representative validation scenarios involved placing a barrier wall at a normalized distance (*x*_*b*_/*L*_*o*_) of 0.2, 0.4, 0.6, 0.8 and 1.0, where the initial SWI wedge length (*L*_*o*_) was 480 m. This yielded a physical barrier distance of 192 m from the sea boundary. The barrier was embedded to a depth of 50 m, resulting in a relative barrier depth (*d*_*b*_/*L*_*a*_) of 0.0167, where *L*_*a*_ equals 3000 m represents the aquifer length.

## Results

### Bayesian optimization process

#### *Optimal hyperparameters*

The optimal hyperparameters obtained from the BO tuning process for the adopted models is presented in Table 3. These hyperparameters were fine-tuned to achieve the best predictive performance for each model, ensuring the highest level of accuracy and efficiency. For the SGB model, the best configuration included 254 estimators and a learning rate of 0.069, signifying a balance between model complexity and learning speed. The maximum tree depth was set to 28, allowing the model to capture intricate relationships in the data, while the minimum samples required for splitting and at leaf nodes were set to 2 and 1, respectively, enabling granular decision-making. Additional hyperparameters such as the regularization parameter (alpha = 0.9) and subsample ratio of 0.524 were tuned to prevent overfitting while maintaining robust performance.

**Table 3:** The optimal hyperparameters obtained from the BO tuning process.

Model	Hyperparameters
SGB	n_estimators = 254; min_samples_leaf = 1; max_depth = 28; alpha = 0.9; min_samples_split = 2; subsample = 0.524; learning_rate = 0.069
XGB	n_estimators = 1908; min_child_weight = 1; max depth = 2; colsample_bytree = 0.660; learning_rate = 0.044; subsample = 0.501
LGB	n_estimators = 171; min_child_samples = 2; max depth = 29; subsample = 0.650; learning_rate = 0.08; colsample_bytree = 0.644; num_leaves = 15
CGB	depth = 5; l2_leaf_reg = 4.137; learning_rate = 0.254

The XGB model achieved its optimal performance with 1908 estimators and a learning rate of 0.044, emphasizing slower but more accurate learning. The maximum tree depth was limited to 2, reflecting its ability to capture simpler interactions within the data. The model also utilized a minimum child weight of 1, a subsample ratio of 0.501, and a column sampling ratio of 0.660, all contributing to a regularized structure that minimizes overfitting and improves generalization.

The LGB model exhibited its best performance with 171 estimators and a learning rate of 0.08, ensuring a balance between computational efficiency and learning precision. The maximum tree depth was set to 29, supported by 15 leaves per tree, allowing for complex decision boundaries. The model also required a minimum of 2 child samples and used a subsample ratio of 0.650 and column sampling ratio of 0.644, which helped maintain model stability and performance in varying conditions.

Finally, the CGB model showed optimal results with a tree depth of 5 and a learning rate of 0.254, indicating a moderate approach to capturing patterns in the data. The l2 regularization parameter was set to 4.137, providing a robust mechanism to control model complexity and mitigate overfitting.

#### *Model performance pre- and post-hyperparameter tuning*

**Fig. 6:**
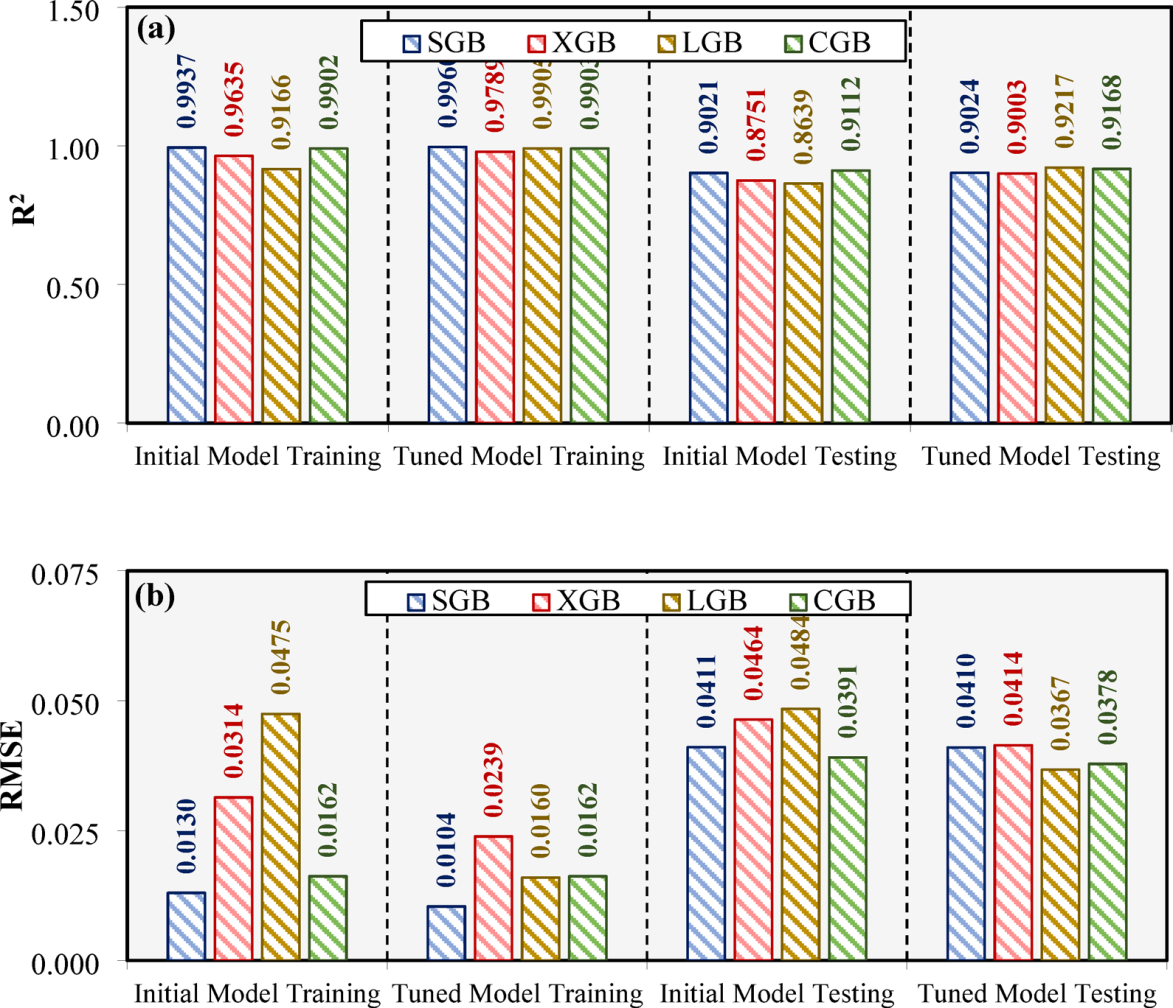
Model performance before and after the BO process across both stages based on (**a**) R^2^ and (**b**) RMSE metrics.

In Fig. 6a, the R^2^ metric is presented for both the initial and tuned models during the training and testing stages. All models exhibit high R^2^ values in the training phase, with slight improvements after the BO process. Among the tuned models, the LGB model consistently demonstrates the highest R^2^ values during both stages, indicating its superior predictive accuracy. The SGB model also performs strongly, with marginally lower R^2^ values compared to the LGB model. The XGB and CGB models display comparatively lower R^2^ values, particularly during the testing stage, suggesting relatively weaker generalization compared to the LGB and SGB models.

Figure 6b illustrates the RMSE values, where lower values indicate better predictive performance. The RMSE values for all models decrease after the BO process, confirming the effectiveness of optimization in reducing error. Similar to the R^2^ results, the LGB model achieves the lowest RMSE values in both the training and testing stages, reinforcing its position as the best-performing model. The SGB model follows closely with competitive RMSE values, while the XGB and CGB models display higher RMSE values, particularly during the testing phase. The reduction in RMSE for all models after tuning highlights the significant impact of the BO process in enhancing their predictive capabilities.

#### *Cross-validation analysis*

**Fig. 7:**
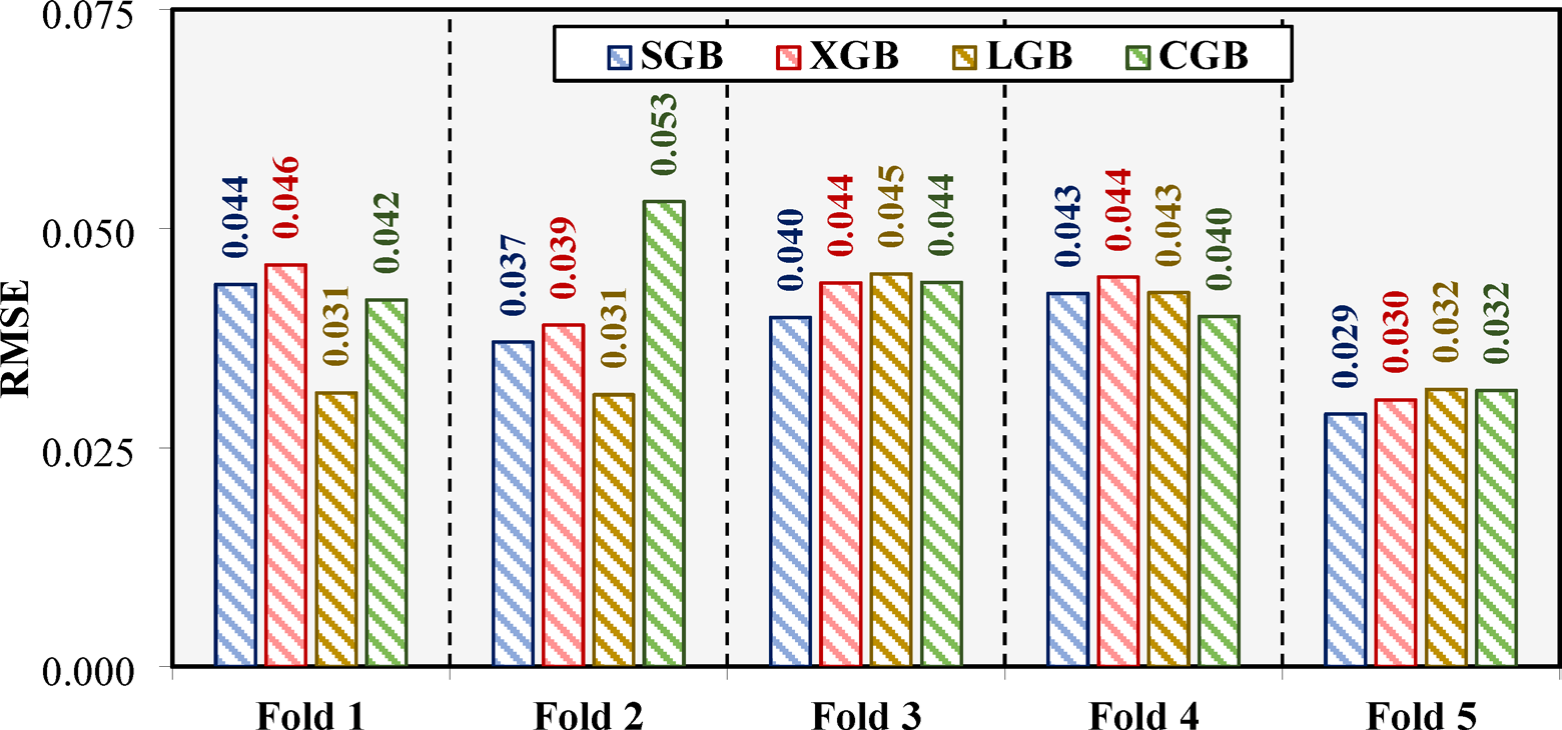
Model performance across the 5-folds during the BO + 5CV process based on the RMSE metric.

The bar chart in Fig. 7 presents the performance of the adopted models across a 5-fold cross-validation process during the Bayesian optimization (BO + 5CV) procedure, using the RMSE metric. Each fold’s results are shown individually, providing insights into the models’ stability and accuracy across different data partitions. For the SGB model, the RMSE values remain consistently low across all folds, with minimal variance. This indicates robust performance and highlights the model’s stability during cross-validation. In comparison, the XGB model exhibits slightly higher RMSE values across most folds, suggesting that its predictions are less accurate and slightly more variable than those of the SGB model.

The LGB model achieves the lowest RMSE values in every fold, demonstrating its exceptional predictive performance and generalization capability during cross-validation. The consistency of its results across the folds further supports its reliability and robustness as the most accurate model among the four. The CGB model, while also performing strongly, shows RMSE values that are competitive but slightly higher than the LGB model’s. This indicates good predictive ability but with marginally higher error rates compared to the LGB model.

Overall, the LGB model appears as the top-performing model, with the lowest RMSE values across all folds. Its stability and accuracy reinforce its suitability for the given task. The SGB model performs reliably but is slightly less accurate than the LGB model. The XGB model shows the highest RMSE values, reflecting lower predictive precision, while the CGB model strikes a balance between strong performance and slightly higher variability compared to the LGB model.

### Model prediction performance assesment

#### *Scatter plots*

**Fig. 8:**
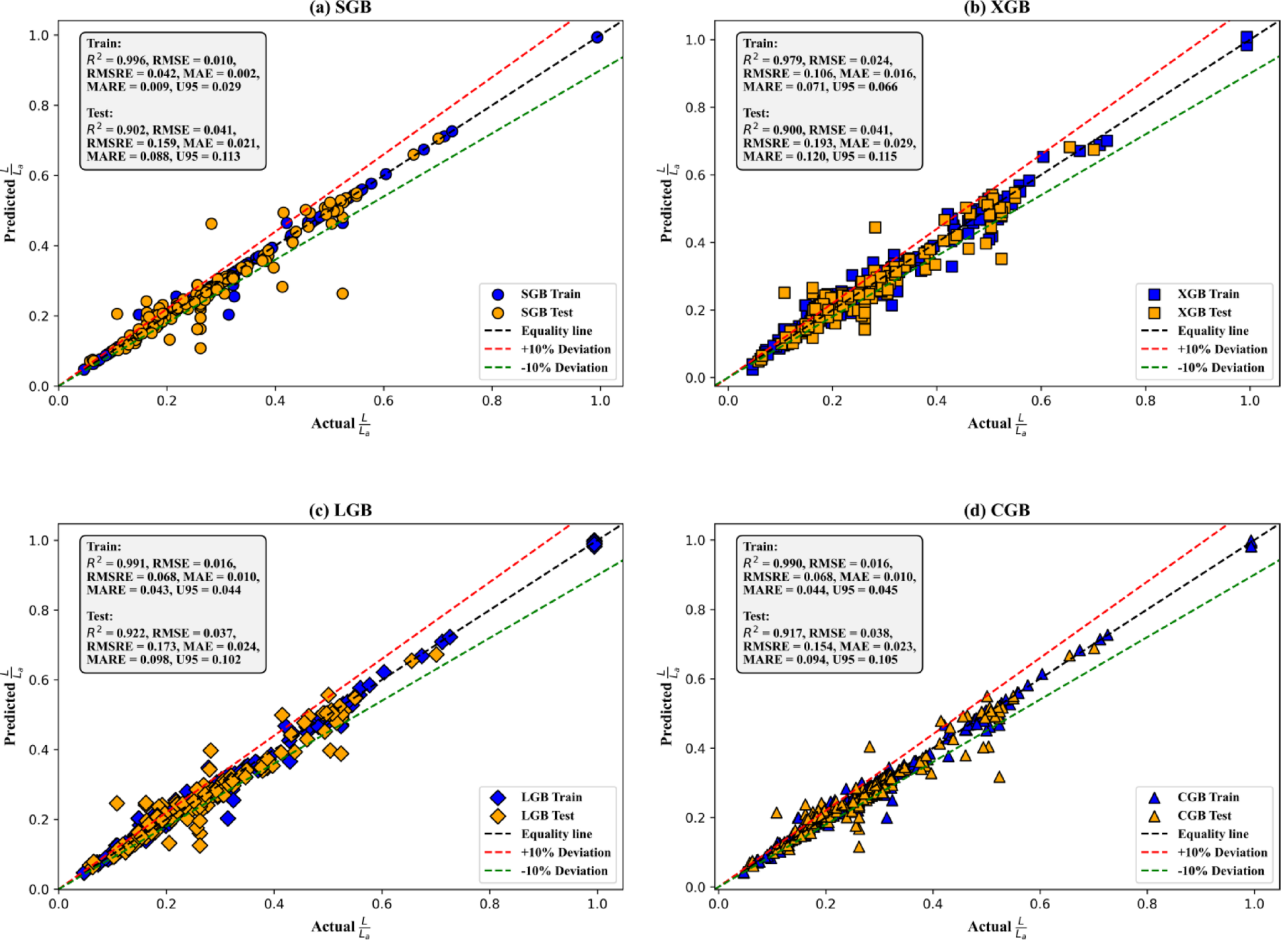
Scatter plots indicating the predictive performance of the adopted models across the training and testing stages (**a**) SGB, (**b**) XGB, (**c**) LGB, and (**d**) CGB.

The scatter plots in Fig. 8 illustrate the predictive performance of the adopted models during both training and testing phases by comparing the predicted and actual values of *L*/*L*_*a*_. The SGB model (Fig. 8a) shows an excellent fit during training, with a high R² value of 0.996 and minimal errors (RMSE: 0.010, RMSRE: 0.042, MARE: 0.009), indicating effective learning. In the testing phase, the R² value slightly decreases to 0.902, with increased errors (RMSE: 0.041, RMSRE: 0.159, MARE: 0.088). This modest drop suggests a limited degree of overfitting, which is a common characteristic of complex ensemble models. However, the overall prediction accuracy remains high, as supported by consistently low error metrics and the stability confirmed through 5-fold cross-validation. These results demonstrate that the SGB model maintains good generalization ability despite minor performance degradation on unseen data. In contrast, the XGB model (Fig. 8b) shows a weaker training fit (R²: 0.979) and slightly higher errors, with comparable performance during testing (R²: 0.900). The LGB model (Fig. 8c) delivers the best overall performance, with strong consistency between training (R²: 0.991, RMSE: 0.016) and testing (R²: 0.922, RMSE: 0.037). Similarly, the CGB model (Fig. 8d) achieves high accuracy (training R²: 0.990, testing R²: 0.917) with minimal performance loss. Across all models, the relatively small gaps between training and testing results, combined with robust cross-validation findings, suggest minimal overfitting and confirm the reliability of the predictive framework.

In terms of uncertainty, the U_95_ values reveal the models’ predictive reliability. The LGB model achieves the lowest U_95_ (0.102) in the testing stage, indicating the least uncertainty and reinforcing its robustness. In contrast, the SGB model shows the highest U_95_ (0.113), suggesting greater variability in its predictions. The XGB model also exhibits high uncertainty (U_95_ = 0.115), further emphasizing its lower predictive reliability. The CGB models strikes a balance with a U_95_ of 0.105, slightly higher than the LGB model but lower than the SGB and XGB models. Overall, the LGB model emerges as the best-performing model due to its superior generalization performance, lowest prediction errors, and minimal uncertainty, making it the most reliable choice for predicting *L*/*L*_*a*_.

#### *REC curves*

**Fig. 9:**
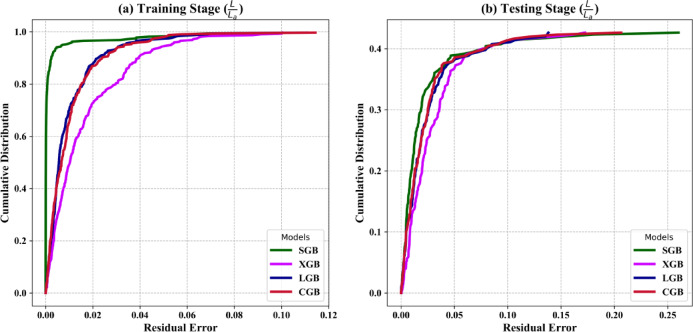
REC curves indicating the cumulative distribution of residual errors across the (**a**) training and (**b**) testing stages in the output predictions by the proposed ML models.

In the training stage (Fig. 9a), the SGB model demonstrates a significantly steep curve, indicating that a high percentage of predictions exhibit minimal residual error. This behavior aligns with its excellent training performance metrics, such as the lowest RMSE (0.010) and RMSRE (0.042), making it the most accurate model during training. The LGB and CGB models follow closely with comparable steepness, reflecting their strong training performance, while the XGB model has the flattest curve, demonstrating higher residual errors and relatively weaker training accuracy.

In the testing stage (Fig. 9b), the LGB model exhibits the steepest REC curve, showing its ability to retain a high proportion of low-error predictions, which corroborates its superior testing metrics, including the highest R^2^ (0.922) and the lowest RMSE (0.037). The CGB model also performs well in testing, with a curve closely following the LGB model, indicating competitive generalization. The SGB model demonstrates slightly reduced performance compared to the LGB and CGB models, with a less steep curve reflecting higher residual errors. The XGB model, once again, has the flattest curve, highlighting its weaker generalization ability and higher distribution of residual errors.

Overall, the REC curves reinforce that the LGB model is the best-performing model in the testing phase, given its ability to consistently predict with low residual errors and its high generalization capability. While the SGB model excels in training, its testing performance is slightly less consistent. The CGB model is robust and competitive but marginally trails the LGB model in generalization. The XGB model remains the weakest model across both training and testing stages, with higher residual errors and less reliable predictions. These curves, combined with quantitative metrics, emphasize the robustness of the LGB model for output prediction.

#### *Violin boxplots*

**Fig. 10:**
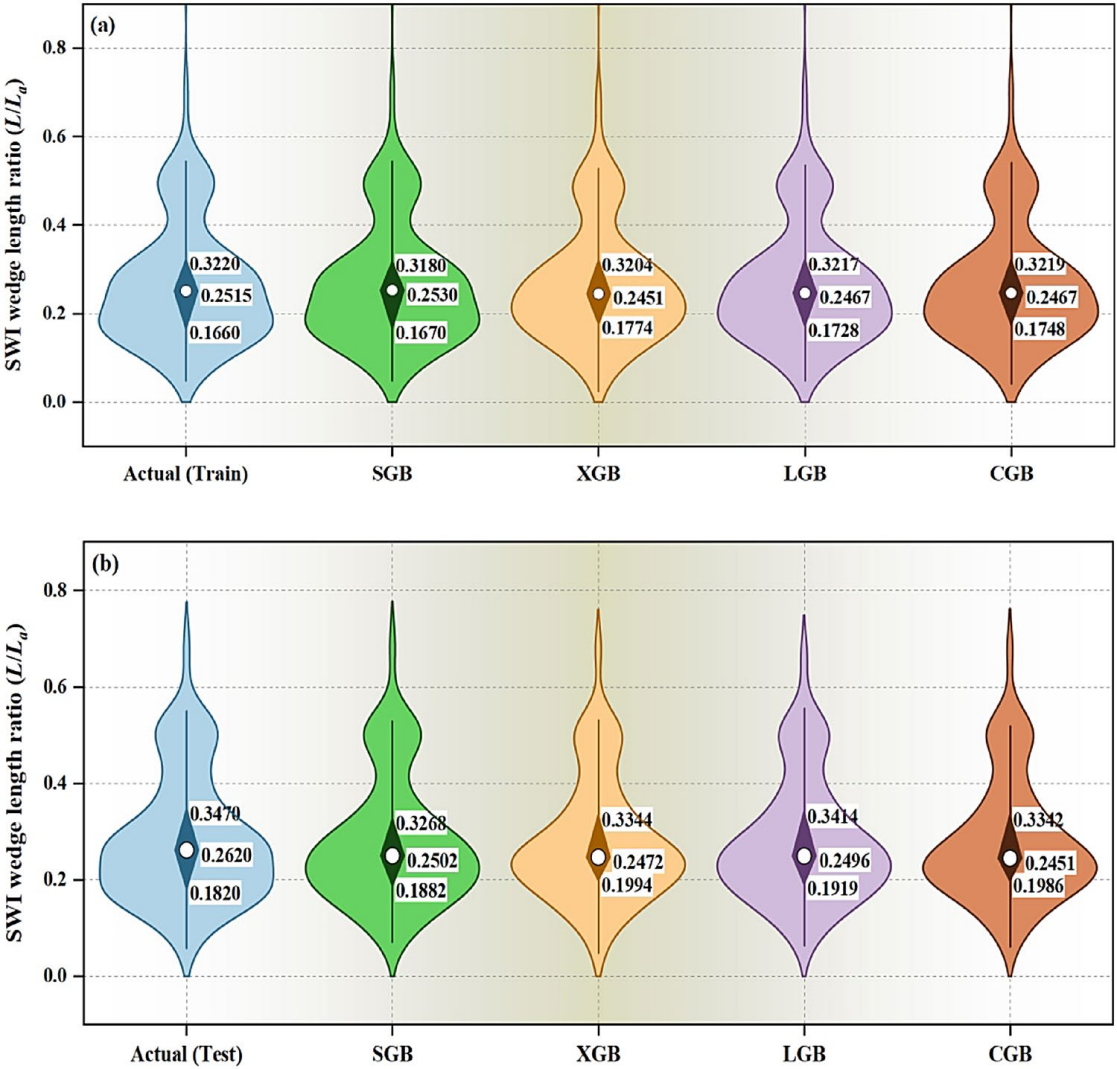
Violin boxplots indicating the predictive performance of the adopted models across the (**a**) training and (**b**) testing stages.

The violin boxplots in Fig. 10 provide a visual summary of the predictive performance of the adopted models. In the training stage (Fig. 10a), the actual data shows a relatively wide distribution of *L*/*L*_*a*_ values with a mean of 0.2515 and a median of 0.3220. The SGB model performs well, closely matching the actual data distribution, with a similar mean (0.2530) and median (0.3180). The XGB model shows a slightly higher variance, as evidenced by the broader violin plot and deviations in its mean (0.2451) and median (0.3204). Both the LGB and CGB models display a strong alignment with the actual data, with the LGB model exhibiting slightly lower variability and a mean (0.2467) closer to the actual distribution compared to the CGB models. This highlights the SGB, LGB, and CGB models as strong contenders during the training phase, with the SGB model achieving the closest fit.

In the testing stage (Fig. 10b), the actual data distribution is slightly shifted compared to training, with a mean of 0.2620 and a median of 0.3470. The SGB model maintains a strong performance with a mean (0.2502) and median (0.3268) closely matching the actual data, though its variability increases slightly. The XGB model again exhibits higher variability, with its mean (0.2472) and median (0.3344) deviating more from the actual distribution. The LGB model demonstrates excellent generalization, with a mean (0.2496) and median (0.3414) closely aligning with the actual values, while also maintaining relatively low variability. The CGB model also performs well, with a mean (0.2451) and median (0.3342) that are similar to the LGB model but exhibit slightly greater variability.

Overall, the LGB model shows the best generalization capability, as evidenced by its consistent alignment with the actual data in both the training and testing stages. Its balanced performance across both phases, along with low variability, makes it the most reliable model. SGB also performs strongly but demonstrates slightly greater variability in the testing stage, while the XGB model exhibits the highest variability, indicating it is less reliable than the other models. The CGB model remains competitive, particularly in testing, but marginally trails behind the LGB model in terms of predictive performance. These findings further reinforce the LGB model as the optimal model for predicting *L*/*L*_*a.*_

###  SHAP analysis

**Fig. 11:**
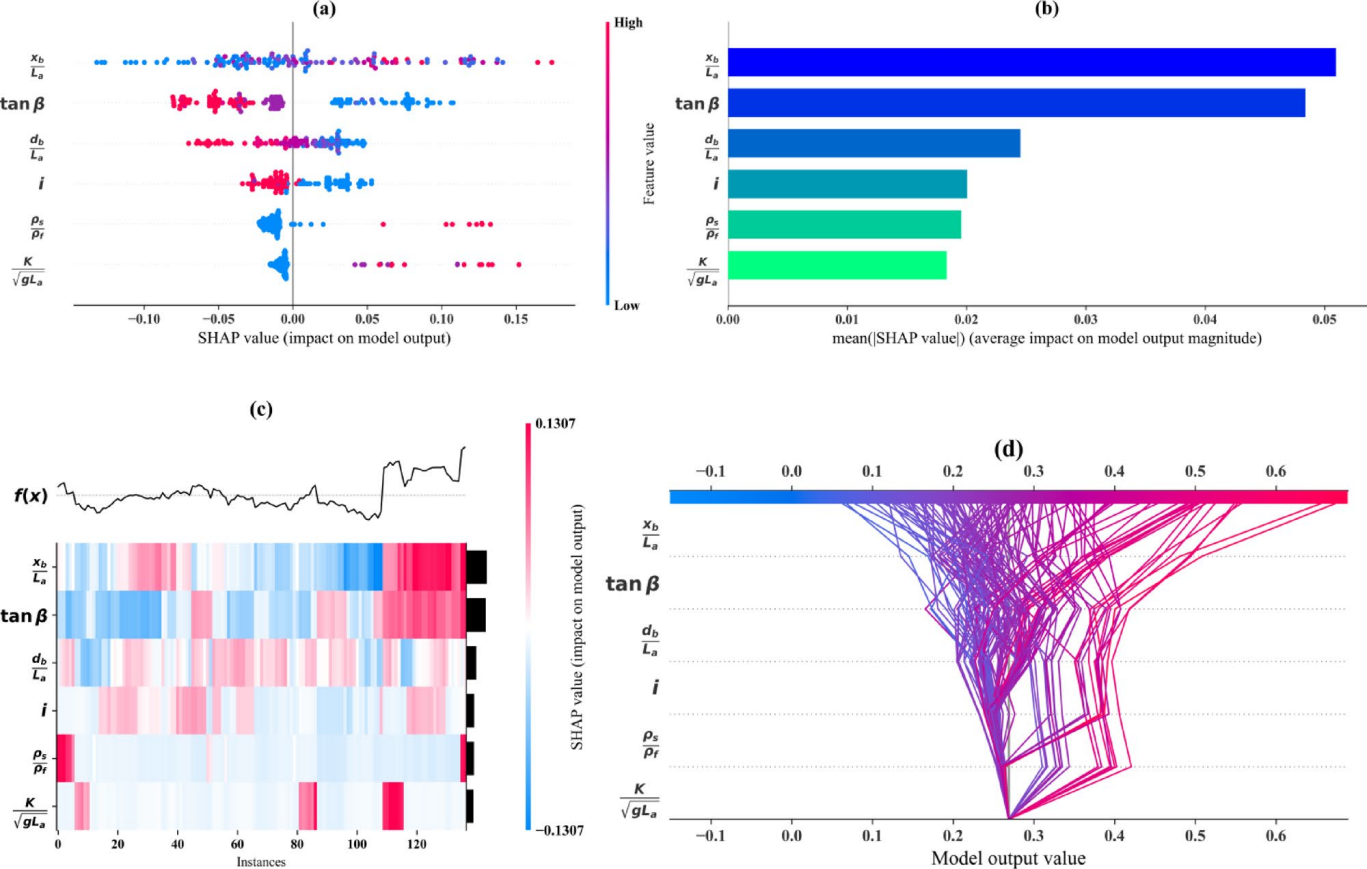
SHAP plots (**a**) summary dot, (**b**) summary bar, (**c**) heatmap, and (**d**) decision river.

Figure 11 presents a comprehensive SHAP analysis to evaluate the feature importance and interpretability of the LGB model predictions. Figure 11a shows the SHAP summary dot plot visualizing the contribution of each feature to the model’s predictions. Each dot represents a single data point, and the color gradient from blue to red indicates the feature value from low to high. The features *x*_*b*_/*L*_*a*_ and *tanβ* have the most significant impact on the predictions, as evident from their wide spread of SHAP values. Features such as *ρ*_*s*_/*ρ*_*f*_ and *K*/(*gL*_*a*_)^½^ have relatively smaller contributions, indicating lower importance.

Figure 11b shows the SHAP summary bar plot ranking the features based on their mean absolute SHAP values, highlighting their average contribution to the model predictions. The features *x*_*b*_/*L*_*a*_ and *tanβ* are confirmed as the most influential, while *ρ*_*s*_/*ρ*_*f*_ and *K*/(*gL*_*a*_)^½^ are less impactful. This ranking provides a concise summary of the relative importance of each feature. Figure 11c shows the SHAP heatmap providing a detailed view of the SHAP values across all instances for each feature. The color scale indicates the magnitude and direction of the SHAP values, with red representing positive contributions and blue indicating negative contributions. The heatmap emphasizes the consistency of the impact of *x*_*b*_/*L*_*a*_ and *tanβ*, while other features exhibit more instance-specific contributions. Figure 11d shows the SHAP decision (river) plot illustrating how individual features cumulatively influence the model’s output for each instance. The features *x*_*b*_/*L*_*a*_ and *tanβ* contribute significantly to driving the predictions closer to the final output. The plot also highlights the interaction between features and how their combined contributions lead to the model’s overall prediction.

###  Model applicabilty

Table 4 presents a comparison between numerical results obtained by Armanuos et al.^27^ and the predicted values from the best-performing ML model (BO-LGB) for *L*/*L*_*a*_ in the Akrotiri coastal aquifer. The predicted values from the BO-LGB model closely align with the numerical results, demonstrating the model’s ability to generalize to real-world cases beyond the training data. To evaluate the prediction accuracy, several performance metrics were calculated. The MAE was 0.039, and the RMSE was 0.0399, indicating a low average deviation between predicted and observed values. In terms of relative accuracy, the model achieved an MARE of 10.08% and RMSRE of 10.12%, reflecting consistent performance across the different barrier configurations. These low error values demonstrate the robustness and practical applicability of the BO-LGB model in estimating *L*/*L*_*a*_ under varying hydrogeological and design conditions. These results highlight the potential of ML approaches to support the analysis and planning of coastal aquifer management strategies involving underground barriers.

**Table 4:** Comparison between numerical results (Armanuos et al.^27^ and predicted values from the best model (BO-LGB) of *L*/*L*_*a*_ for the Akrotiri coastal aquifer, including relevant input parameters.

Inputs						Output: *L*/*L*_*a*_	
*tanβ*	*i*	*ρ*_*s*_/*ρ*_*f*_	*K*/(*gL*_*a*_)^1/2^	*d*_*b*_/*L*_*a*_	*x*_*b*_/*L*_*a*_	Num^27^	BO-LGB
0.017	0.002	1.036	4.53 × 10^− 5^	0.008	0.160	0.455	0.406
					0.128	0.445	0.404
					0.096	0.395	0.350
					0.064	0.365	0.330
					0.032	0.265	0.240

### Graphical user interface

The interactive graphical user interface (GUI) was implemented utilizing Python’s Tkinter library, a commonly adopted framework for developing user-friendly and responsive desktop applications^56^. Tkinter enables the integration of input fields, control buttons, and dynamic outputs, making it ideal for developing engineering tools that require rapid calculations and user engagement. Its simplicity and flexibility support the deployment of accurate, real-time predictive models in an accessible format for end users^57–60^.

**Fig. 12:**
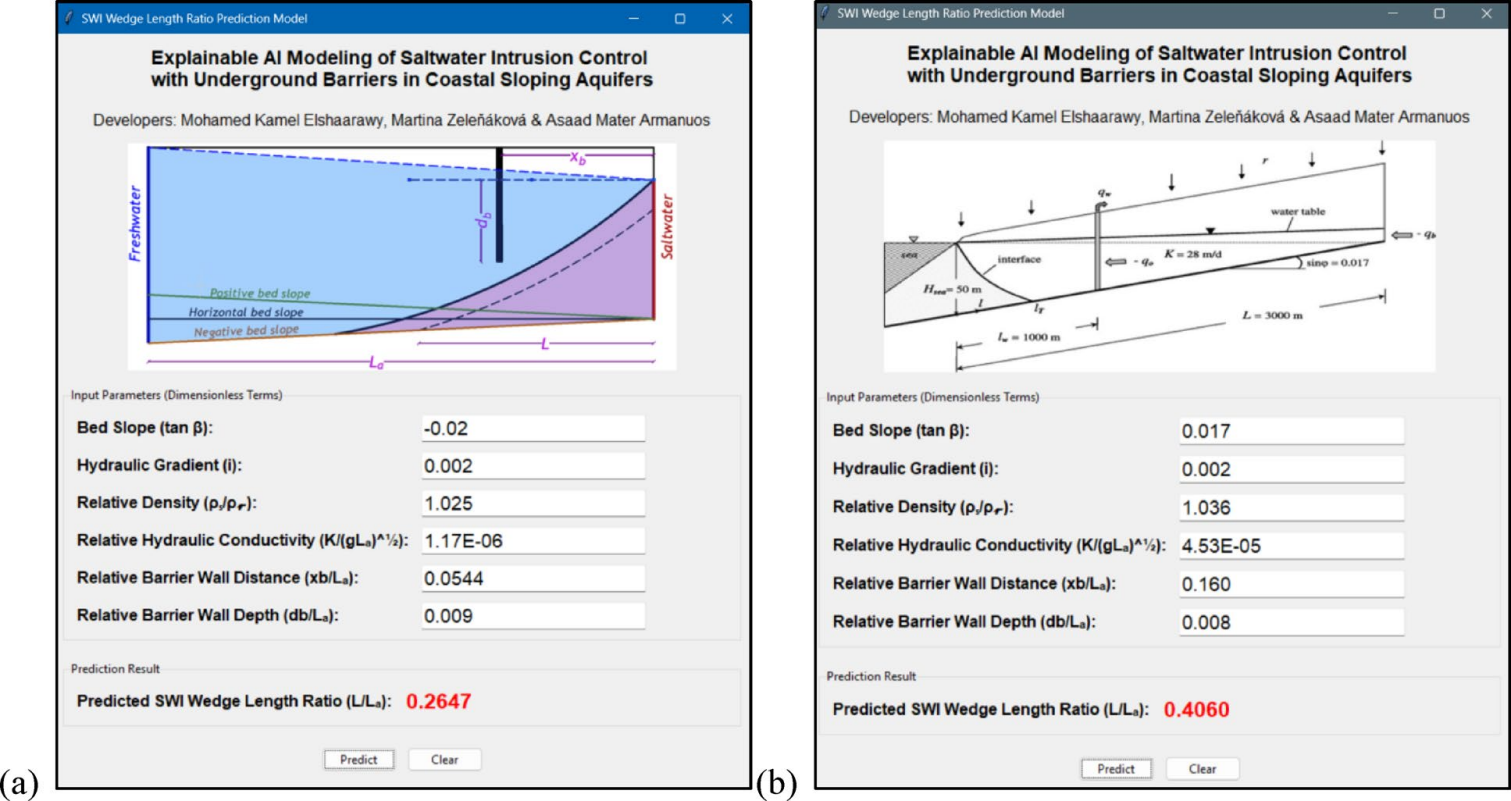
Developed GUI for predicting *L*/*L*_*a*_ in coastal sloping aquifers with underground barriers: (**a**) example using a case from the collected database (**b**) application to the Akrotiri coastal aquifer with embedded underground barriers.

The developed GUI enables users to input normalized hydrogeological parameters and underground barrier characteristics and instantly obtain the predicted *L*/*L*_*a*_. This tool supports decision-making in coastal groundwater management by offering a fast and reliable way to estimate SWI behavior without the need for complex numerical modeling. Figure 12a presents an example use of the GUI based on a case from the collected dataset. The visual layout shows clearly labeled input fields for each parameter, with the resulting predicted value of *L*/*L*_*a*_ displayed at the bottom. This example highlights the GUI’s functionality and ease of use, demonstrating how predictions can be obtained with minimal user effort.

Figure 12b shows the GUI applied to the Akrotiri coastal aquifer, a real-world case study. The input values correspond to parameters derived from SEAWAT simulations by Armanuos et al.^27^. Once entered, the GUI produces a predicted *L*/*L*_*a*_ that closely aligns with the original numerical simulation result. This agreement illustrates the reliability of the underlying ML model and confirms the GUI’s practical applicability for real-case evaluation and planning. Together, these figures demonstrate both the predictive capability and usability of the developed interface, reinforcing its value as a decision-support tool in the context of saltwater intrusion control in sloping coastal aquifers.

## Discussion

### Influence of Bayesian Optimization on hyperparameter tuning

The implementation of Bayesian Optimization (BO) significantly enhanced the performance of all adopted gradient boosting models by systematically exploring hyperparameter spaces to identify optimal configurations. As detailed in Table 3, the hyperparameter tuning process led to marked improvements in model generalization and predictive accuracy. For example, the LGB model achieved optimal performance with a learning rate of 0.08 and a tree depth of 29, allowing it to learn complex relationships without overfitting. Similarly, the SGB model, with 254 estimators and carefully tuned regularization parameters (e.g., alpha = 0.9), balanced learning capacity and noise control. The use of BO enabled each model to minimize prediction error, as reflected in reduced RMSE and improved R² values during both training and testing. These outcomes underscore the effectiveness of the BO process as a hyperparameter tuning strategy for optimizing model robustness, reducing variability, and enhancing generalization^61–63^.

### Improvements in predictive accuracy and model robustness

The performance improvements resulting from the BO method were clearly evident across all evaluated metrics, as presented in Figs. 6, 7, 8, 9 and 10. The LGB model consistently achieved the highest R² values and lowest RMSE across both stages, confirming its superior predictive capability. Notably, the slight decline in performance from training (R^2^=0.991) to testing (R^2^=0.922) was minimal and supported by consistently low RMSE values (0.016 and 0.037, respectively), indicating strong generalization and limited overfitting. The 5-fold cross-validation analysis (Fig. 7) highlighted the model’s reliability, with low and consistent RMSE across all folds. Additionally, the REC curves (Fig. 9) reinforced this finding, with the LGB model demonstrating the steepest curve in the testing stage, indicative of accurate, low-residual predictions. These results confirm that the BO method not only improved accuracy but also enhanced stability across different data partitions. The SGB and CGB models also performed strongly, though slightly trailing the LGB model, while the XGB model exhibited the highest residuals and weakest generalization. This consistency highlights its ability to generalize well to unseen data. The comparison between pre- and post-tuning performance across all models emphasizes the value of hyperparameter optimization in minimizing prediction errors and improving generalization capabilities^64–66^.

### Interpretability and feature contributions

Interpretability is essential for the adoption of ML models in engineering practice, and this study addressed that need using the SHAP technique. As shown in Fig. 11, the SHAP analysis provided a detailed understanding of feature influence on model predictions. Features such as normalized barrier wall location (*x*_*b*_/*L*_*a*_) and bed slope (*tanβ*) emerged as the most influential predictors of the wedge length ratio (*L*/*L*_*a*_), aligning with physical expectations of SWI behavior in sloping aquifers. The SHAP dot and bar plots (Fig. 11a and b) confirmed their dominant contributions, while the heatmap and decision plots (Fig. 11c and d) illustrated their consistent and cumulative effects across instances.

These findings corroborate earlier work by Armanuos et al.^27^ where they identified seaward barrier placement and steeper slopes as key factors in SWI control. However, while they relied on numerical modeling (SEAWAT) to infer these behaviors, this study leverages SHAP to extract them directly from the ML model, improving interpretability and enabling quantification of feature effects. For instance, the SHAP results emphasize a particularly strong response of *L*/*L*_*a*_ to changes in *x*_*b*_/*L*_*a*_ between 0.1 and 0.2, providing actionable insights for coastal aquifer design. This integration of ML and explainable AI thus enhances both model transparency and its relevance for real-world applications^67–69^.

###  Practical applications through GUIs

To translate the theoretical and predictive strengths of the proposed ML framework into actionable tools, a GUI was developed using Python’s Tkinter library. The GUI enables users to input normalized hydrogeological and design parameters to instantly predict the *L*/*L*_*a*_ ratio, supporting efficient and informed decision-making in SWI mitigation. The tool offers intuitive usability while retaining the model’s robustness and accuracy, thereby lowering the technical barrier for adoption among engineers and water managers^58–60^.

Importantly, the practical applicability of the proposed ML model was validated using the Akrotiri coastal aquifer, a realistic benchmark case derived from numerical simulations by Armanuos et al.^27^. The predictions of the BO-LGB model closely matched numerical outputs, with an RMSE of 0.0399 and an MAE of 0.039 (Table 4), confirming the model’s ability to generalize beyond the training data. This alignment between simulated and predicted values across different configurations reinforces the utility of the GUI as a reliable tool for planning underground barrier systems in sloping coastal aquifers^70^.

##  Conclusions

SWI remains a pressing issue in the sustainable management of coastal aquifers, particularly in regions where freshwater resources are under increased pressure due to human activities and climate change. Accurately predicting *L*/*L*_*a*_​ is critical for implementing effective mitigation strategies. This study applied the BO tuning method to enhance the performance of gradient boosting models: SGB, XGB, LGB, and CGB using a dataset of 456 samples characterized by key dimensionless input variables, such as bed slope, hydraulic gradient, and barrier wall properties. Summing up the results, the following conclusions can be drawn:

- The BO-LGB model demonstrated the best overall performance, achieving the lowest RMSE values and the highest R^2^ scores during both the training and testing stages. It also exhibited minimal predictive uncertainty, as reflected in its U_95_ values, making it the most robust and reliable model for predicting *L*/*L*_*a*_​​.
- The BO-SGB model also performed strongly, particularly during the training phase, where it achieved results comparable to the LGB model. However, its performance was slightly less consistent during testing, with marginally higher errors and variability.
- The BO-CGB model provided competitive results with reasonable RMSE values and high R^2^ scores. While it trailed the LGB model slightly in accuracy and generalization, its results indicated good predictive capabilities.
- The BO-XGB model, although less accurate than the other models, demonstrated acceptable predictive performance but had the highest residual errors and variability, particularly during testing.
- The SHAP analysis identified *x*_*b*_/*L*_*a*_ and *tanβ* as the most influential features driving the model’s predictions, followed by *d*_*b*_/*L*_*a*_ and *i*. Parameters like *ρ*_*s*_/*ρ*_*f*_ and *K*/(*gL*_*a*_)^½^ had lower relative importance.
- An interactive GUI was developed to enhance the accessibility and usability of the predictive models, allowing users to input parameters and receive predictions in a user-friendly format. The GUI facilitates scenario-based analysis, making it a practical tool for researchers and decision-makers working on coastal aquifer management.
- The developed models were validated using the Akrotiri coastal aquifer case study, showing excellent agreement with SEAWAT simulation results with RMSE of 0.04, thereby confirming the models’ real-world applicability.

Despite the strong performance of the proposed ML models, this study primarily relied on numerically simulated datasets derived from controlled scenarios. While such data offer consistency and scalability, they may not fully capture the complex spatial variability, geological heterogeneity, and temporal dynamics present in natural aquifer systems. However, to enhance the practical relevance and credibility of the models, the predictions were externally validated against simulation results from the Akrotiri coastal aquifer in Cyprus. This validation step demonstrated strong agreement and confirmed the models’ robustness in replicating real-world SWI behavior under diverse hydrogeological conditions.

Future research should build on this foundation by incorporating comprehensive field-measured datasets, including time-series monitoring and multi-layered aquifer systems. Additionally, expanding the framework to include multi-objective optimization and uncertainty propagation can further improve model generalizability and resilience. Overall, this study offers a robust and interpretable ML-based framework that combines theoretical rigor with practical applicability, paving the way for more data-driven and accessible tools in coastal aquifer management.

## Data Availability

Data, models, or codes that support the findings of this study are available from the corresponding author upon reasonable request.
